# Comprehensive analysis of transient receptor potential channels-related signature for prognosis, tumor immune microenvironment, and treatment response of colorectal cancer

**DOI:** 10.3389/fimmu.2022.1014834

**Published:** 2022-10-18

**Authors:** Lei Wang, Xingte Chen, Hejun Zhang, Liang Hong, Jianchao Wang, Lingdong Shao, Gang Chen, Junxin Wu

**Affiliations:** ^1^ Department of Radiation Oncology, Clinical Oncology School of Fujian Medical University, Fujian Cancer Hospital, Fuzhou, China; ^2^ Department of Pathology, Clinical Oncology School of Fujian Medical University, Fujian Cancer Hospital, Fuzhou, China

**Keywords:** transient receptor potential channels, colorectal cancer, prognosis, immune checkpoint inhibitor, neoadjuvant treatment

## Abstract

**Background:**

Transient receptor potential channels (TRPC) play critical regulatory functions in cancer occurrence and progression. However, knowledge on its role in colorectal cancer (CRC) is limited. In addition, neoadjuvant treatment and immune checkpoint inhibitors (ICIs) have increasing roles in CRC management, but not all patients benefit from them. In this study, a TRPC related signature (TRPCRS) was constructed for prognosis, tumor immune microenvironment (TIME), and treatment response of CRC.

**Methods:**

Data on CRC gene expression and clinical features were retrospectively collected from TCGA and GEO databases. Twenty-eight TRPC regulators (TRPCR) were retrieved using gene set enrichment analysis. Different TRPCR expression patterns were identified using non-negative matrix factorization for consensus clustering, and a TRPCRS was established using LASSO. The potential value of TRPCRS was assessed using functional enrichment analysis, tumor immune analysis, tumor somatic mutation analysis, and response to preoperative chemoradiotherapy or ICIs. Moreover, an external validation was conducted using rectal cancer samples that received preoperative chemoradiotherapy at Fujian Cancer Hospital (FJCH) *via* qRT-PCR.

**Results:**

Among 834 CRC samples in the TCGA and meta-GEO cohorts, two TRPCR expression patterns were identified, which were associated with various immune infiltrations. In addition, 266 intersected genes from 5564 differentially expressed genes (DEGs) between two TRPC subtypes, 4605 DEGs between tumor tissue and adjacent non-tumor tissue (all FDR< 0.05, adjusted P< 0.001), and 1329 prognostic related genes (P< 0.05) were identified to establish the TRPCRS, which was confirmed in the TCGA cohort, two cohorts from GEO, and one qRT-PCR cohort from FJCH. According to the current signature, the high-TRPC score group had higher expressions of PD-1, PD-L1, and CTLA4, lower TIDE score, and improved response to anti-PD-1 treatment with better predictive ability. Compared to the high-TRPC score group, the low-TRPC score group comprised an immunosuppressive phenotype with increased infiltration of neutrophils and activated MAPK signaling pathway, but was more sensitive to preoperative chemoradiotherapy and associated with improved prognosis

**Conclusions:**

The current TRPCRS predicted the prognosis of CRC, evaluated the TIME in CRC, and anticipated the response to immune therapy and neoadjuvant treatment.

## Introduction

Colorectal cancer (CRC) is one of the most common cancers worldwide (1), with 151,030 cases diagnosed annually in the United States (2). Currently, its incidence is increasing worldwide (1). Moreover, CRC is the third leading cause of cancer mortality worldwide (1), with 0.9 million deaths in the United States yearly. Current plights of the CRC are as followed: 1) lack of specific markers of early screening, regardless of promotion of colonoscopy (3); 2) inaccuracy of the current staging systems on prognosis and management (4, 5), and 3) short of biomarkers for both local and systematic treatment in the era of precision medicine and individualized therapy (4, 5). Hence, early diagnosis biomarkers, accurate prognosis prediction, and precise direct management for CRC are urgently required (3).

Transient receptor potential channels (TRPC) was first reported in 1969 (6). Numerous homologous TRPC family genes are identified as TRPC regulators (TRPCR) (7, 8). In 2021, Ardem Patapoutian and David Julius were awarded the Nobel Prize in Physiology or Medicine for the discovery of TRPC (9), which are multifunctional signaling molecules investigated in channelopathy-related diseases including neurodegenerative (10), cardiovascular (11), and metabolic diseases (12). However, increasing reports support their roles in carcinogenesis, tumor invasion, migration, angiogenesis, and prognosis (13–15). There were differences in expression of several TRPCR, such as TRPV1, TRPV6, TRPM4, and TRPC6 between CRC and normal tissues (16, 17). Some TRPCR, such as TRPM6 and TRPC1, are associated with the prognosis of patients with CRC (16, 17). However, a comprehensive analysis of TRPCR on CRC prognosis and management is inadequate.

Although neoadjuvant treatment and immune checkpoint inhibitors (ICIs) play an increasing role in CRC management, not all patients benefit from them (18, 19). In addition, no biomarkers exist to screen their potential benefit (18). Evidence showed that tumor immune microenvironment (TIME) (20), which is associated with TRPC *via* polarization of macrophages, recruitment of chemokines, and activation of effector cells, strongly influences cancer treatment response (20, 21). In the present study, non-negative matrix factorization (NMF) clustering was adopted to identify the correlations between TRPCR and immune infiltration, and a TRPC-related signature (TRPCRS) was established to predict the prognosis of CRC, and explore the intrinsic connections between TRPCRS and TIME. Furthermore, correlations between TRPCRS and response to neoadjuvant treatment or ICIs were conducted to determine the potential value of the current signature.

## Methods

### Patients and data source

**Figure 1:**
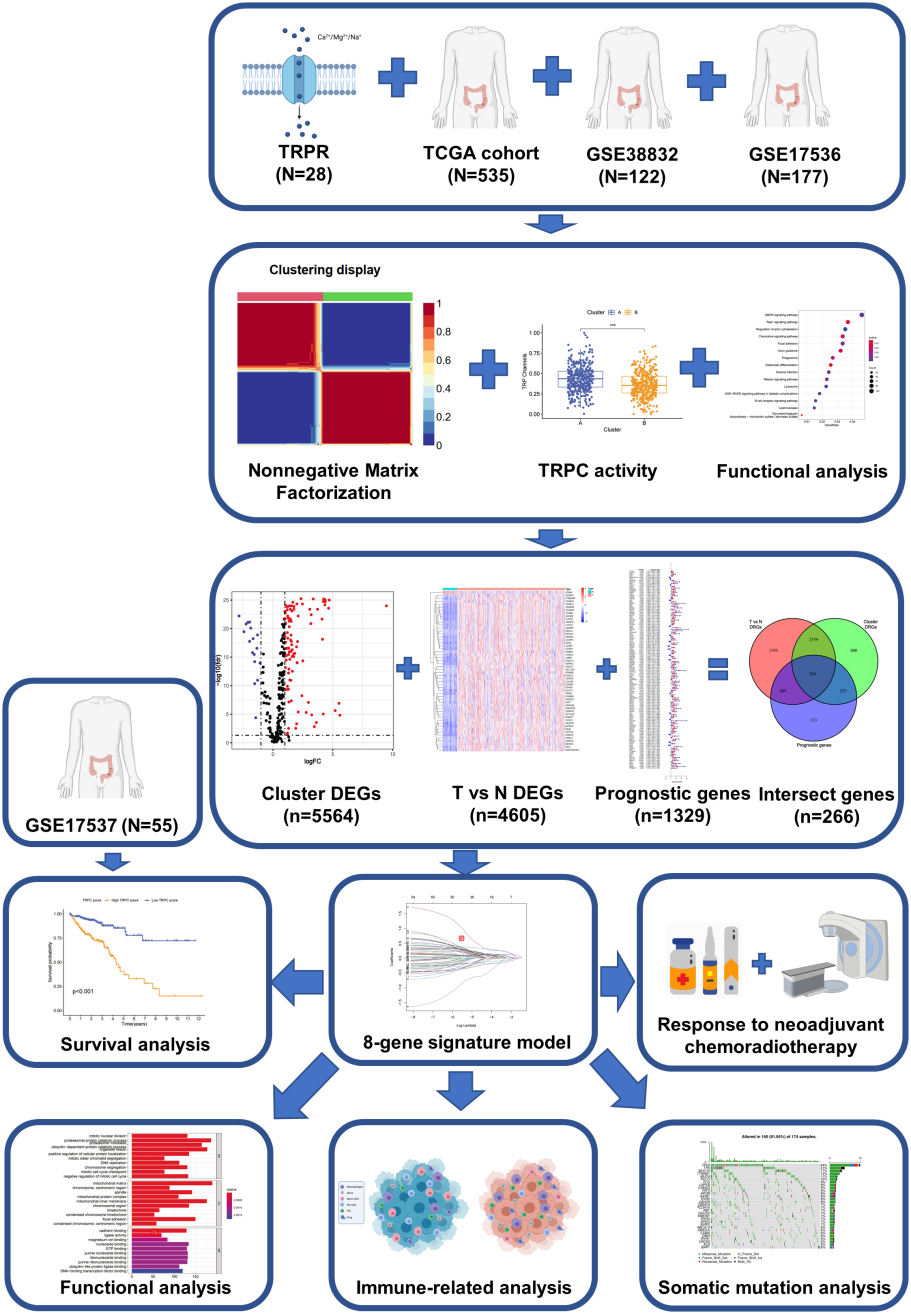
The flow-chart of this study.

The analytical process of the study is provided in **Figure 1**. Data on CRC gene expression and clinical features were retrospectively collected from The Cancer Genome Atlas (TCGA, https://cancergenome.nih.gov/) and Gene Expression Omnibus (GEO) databases (https://www.ncbi.nlm.nih.gov/geo/). TCGA RNA sequencing data were converted from fragments per kilobase of exon model per million mapped fragments (FPKM) format to millions of transcripts per kilobase (TPM). Batch effects among TCGA-COAD, TCGA-READ, and GEO datasets were eliminated using “ComBat” method in “sva” R package, and TCGA-COAD-READ and meta-GEO (GSE38832 (22) and GSE17536 (23)) datasets were constructed. Genomic mutation data of TCGA-COAD-READ, including somatic mutations and copy number variations, were obtained from UCSC’s Xena database. Copy number changes of 28 TRPCR in human chromosomes were mapped using the R package “rcircos”. The corresponding TRPCR were extracted from the Gene Set Enrichment Analysis (GSEA) website (https://www.gsea-msigdb.org/GSEA/index.jsp, **Table S1**). Moreover, 85 frozen rectal cancer samples that received both, neoadjuvant treatment and radical surgery, at Fujian Cancer Hospital (FJCH) between March 2016 - March 2021 were added to conduct external validation. This study was approved by the Ethics Committee of FJCH (K2021-03-017). The baseline characteristics of patients in the cohort are presented in **Table 1**.

**Table 1:** Clinical characteristics of the CRC patients used in this study.

	TCGA-COAD-READ cohort	Meta-GEO cohort		GSE17537 cohort	GSE45404 cohort	GSE87211 cohort	FJCH cohort
		GSE38832 cohort	GSE17536 cohort				
**No. of patients**	535	122	177	55	80	203	85
**Age**							
≤65	297 (55.5%)	NA	83 (46.9%)	33 (60.0%)	49 (61.3%)	128 (63.1%)	65 (76.5%)
>65	236 (44.1%)	NA	94 (53.1%)	22 (40.0%)	31 (38.7%)	74 (36.4.0%)	20 (23.5%)
unknown	2 (0.4%)	NA	0 (0.0%)	0 (0.0%)	0 (0.0%)	1 (0.5%)	0 (0.0%)
**Gender**							
Female	259 (48.4%)	NA	81 (45.8%)	29 (52.7%)	31 (38.7%)	61 (30.0%)	30 (35.3%)
Male	265 (49.5%)	NA	96 (54.2%)	26 (47.3%)	49 (61.3%)	142 (70.0%)	55 (64.7%)
unknown	11 (2.1%)	NA	0 (0.0%)	0 (0.0%)	0 (0.0%)	0 (0.0%)	0 (0.0%)
**Stage**							
I	96 (18.0%)	18 (14.7%)	24 (13.6%)	4 (7.3%)	NA	0 (0.0%)	0 (0.0%)
II	191 (35.7%)	35 (28.7%)	57 (32.2%)	15 (27.3%)	NA	63 (31.0%)	43 (50.6%)
III	150 (28.0%)	39 (32.0%)	57 (32.2%)	19 (34.5%)	NA	125 (61.6%)	42 (49.4%)
IV	76 (14.2%)	30 (24.6%)	39 (22.0%)	17 (30.9%)	NA	12 (5.9%)	0 (0.0%)
unknown	22 (4.1%)	0 (0.0%)	0 (0.0%)	0 (0.0%)	NA	3 (1.5%)	0 (0.0%)
**Grade**							
Grade 1	NA	NA	16 (9.0%)	NA	NA	NA	2 (9.4%)
Grade 2	NA	NA	134 (75.7%)	NA	NA	NA	74 (87.1%)
Grade 3	NA	NA	27 (15.3%)	NA	NA	NA	2 (9.3%)
Grade 4	NA	NA	0 (0.0%)	NA	NA	NA	3 (3.5%)
unknown	NA	NA	NA	NA	NA	NA	4 (4.7%)
**MSI**							
MSI-H	60 (11.2%)	NA	NA	NA	NA	NA	0 (0.0%)
MSI-L	72 (13.5%)	NA	NA	NA	NA	NA	0 (0.0%)
MSS	287 (53.6%)	NA	NA	NA	NA	NA	49 (57.6%)
unknown	116 (21.7%)	NA	NA	NA	NA	NA	36 (42.4%)
**KRAS status**							
Mutation	NA	NA	NA	NA	NA	84 (41.4%)	8 (9.4%)
WT	NA	NA	NA	NA	NA	109 (53.7%)	17 (20.0%)
unknown	NA	NA	NA	NA	NA	10 (4.9%)	60 (70.6%)
**Response to neoadjuvant chemoradiotherapy**							
Response	NA	NA	NA	NA	35 (43.8%)	NA	45 (52.9%)
Non-response	NA	NA	NA	NA	45 (56.2%)	NA	40 (47.1%)

NA, Not available.

### Consensus molecular clustering of 28 TRPCR

Different TRPCR expression patterns were identified using NMF based on the expressions of 28 TRPCR using the R package “NMF” (version 0.22.0) (24). The expressions of 28 TRPCR (matrix A) were decomposed into two non-negative matrices, W and H (i.e., A≈WH). Matrix A was repeatedly decomposed, and its output was aggregated to obtain a consistent cluster of CRC samples (TCGA-COAD-READ and meta-GEO). The optimal k of clusters was selected according to apparent, discrete, and silhouette coefficients. Brunet algorithm and 200 nrun were used for consensus clustering.

### Identification of differentially expressed genes and enriched pathways

Differentially expressed genes (DEGs) among TRPC subtypes (DEG_a_, false discovery rate< 0.05, adjusted P< 0.001) were obtained using the “limma” R package. The pathway activity of “REACTINE_TRP_CHANNELS” in each sample was calculated using “GSVA” packages, and the differences among various TRPC subtypes were analyzed.

### Construction of TRPCRS

DEGs between tumor tissues and adjacent non-tumor tissues (DEGb, FDR< 0.05, adjusted P< 0.001) were determined using the “limma” R package. Prognostic genes were screened using the “survival” R package *via* univariate Cox regression analysis (P< 0.05). The overlapped genes among DEGa, DEGb, and prognostic genes were identified as candidate TRPC-related genes (TRPCGs). These candidate genes were screened again based on the least absolute shrinkage and selection operator (LASSO) (25) estimation to avoid over-fitting the model. The optimal value of the penalty coefficient lambda was selected after running the cross-validation probability 1000 times through the “glmnet” software package. Considering that the genes included in the gene signature were derived from DEGa between two clusters with significantly different TRPC, the resulting gene signature was called TRPCRS. Thus, the equation was established as follows:


$$TRPC\;score=\sum_{i=1}^{n}Coef(i)\times x(i)$$

According to the corresponding median of TRPCRS in each dataset, patients were divided into low TRPC score and high TRPC score groups.

### Validation of TRPC score model

The TRPC score was validated in TCGA-COAD-READ, meta-GEO, and an external validation set of GSE17537 (26). The survival difference between the two groups was visually displayed using the receiver operator characteristic (ROC) curves and “survival” R package. The significance of the TRPC score was further analyzed using the multivariate Cox regression model, and the relationships between the TRPC score and clinical features were evaluated using the Wilcox test.

### Function analysis

The Gene Ontology (GO) and Kyoto Encyclopedia of Genes and Genomes (KEGG) enrichment analyses were conducted on DEG_a_ using the “clusterProfiler” R package (27). Stromal and immune cells infiltrated in malignant tumors were estimated using the ESTIMATE algorithm, utilizing the unique properties of transcription profiles to infer tumor cell count and tumor purity.

### Immune-related analysis

The relative abundance of 28 immune cells in TIME was evaluated using the “GSVA” package (28). Differences in the immune cells and immune checkpoint genes were compared between high- and low-TRPC scores. Scores of tumor immune dysfunction and exclusion (TIDE), microsatellite instability (MSI) expression, dysfunction, and rejection were calculated using http://tide.harvard.edu, and the differences were compared between the two groups. In addition, the prediction value of the TRPC score for immunotherapy was estimated using the “IMvigor 210” dataset package (29).

### Somatic mutation analysis

Quantity and quality of mutations in high- and low- TRPC scores were calculated using the “Maftools” R package. Missense, nonsense, continuous and silent, and frameshift/in-box insertion and deletion mutations were counted after excluding germline mutations without somatic mutations. Tumor mutation burden (TMB) is defined as the total number of somatic mutations.

### Development of risk prediction model

In addition to TRPC scores and clinical features, a nomogram predicting 1-year, 3-year, and 5-year overall survival (OS) of patients with CRC was established using the “RMS” R package. The nomogram prediction was evaluated using the calibration curve, restricted mean survival (RMS), C index, ROC curve, and decision curve analysis (DCA).

### Response prediction of neoadjuvant therapy

GSE45404 (30) and GSE87211 (31) were administrated to conduct external validation, among which patients with rectal cancer received neoadjuvant treatment. GSE45404 contained data on response to neoadjuvant treatment and was graded using the Mandard tumor regression grade (TRG), while GSE87211 contained data on clinicopathological characteristics and survival. ”REACTINE_TRP_CHANNELS”, “KEGG_MISMATCH_REPAIR”, “KEGG_MAPK_SIGNALING_PATHWAY”, and “KEGG_B_CELL_SIGNALING_PATHWAY” of each sample were calculated using “GSVA” R package, and the infiltration situation of 28 immune cells in the TIME were plotted using “ggplot2” and “corrplot” R packages, respectively.

### Quantitative real time polymerase chain reaction

Quantitative real time polymerase chain reaction (qRT-PCR) was performed on 85 samples by the Department of Pathology Department of FJCH. RNA was extracted using TRIzol (Takara, Kusatsu, Shiga, Japan), and random primers were reverse transcribed using a cDNA synthesis kit (Thermo Fisher Scientific, Waltham, MA, USA). In addition, mRNA expression levels were detected using Roche LightCycler 480 (Basel, Switzerland) and FastStart Essential DNA Green Master Mix (Thermo Fisher Scientific). The mRNA expressions of each hub gene were normalized to that of β-actin. All qRT-PCR analyses were conducted in triplicates, and the average value was calculated using the Livak method. The primers used in this study were synthesized using Sunya Biotech (Fuzhou, China) and are listed in **Supplementary Table 2**.

### Statistical analyses

All analyses in this study were performed using R-3.6.1. Normally distributed variables were compared using Student’s t-test, while non-normally distributed variables were compared using the Wilcoxon rank sum test. All tests were two-sided, and P< 0.05 was considered to be statistically significant.

## Results

### Genetic alteration landscape of TRPCR in CRC

In the present study, TRPCR were widely located in almost all human chromosomes (**Supplementary Figure 1A**). **Supplementary Figure 1B** depicts interactions of 28 TRPCR expressions, and TRPC1, TRPA1, and RIPK as the top three TRPCR. Furthermore, analysis of 28 TRPCR revealed that copy number variations (CNV) mutations were prevalent. TRPC4AP, TRPC4, TRPA1, TRPV6, TRPC1, TRPC5, TRPV3, TRPV1, and TRPM6 showed widespread CNV amplification. In contrast, TRPV5, MCOLN3, TRPM8, RIPK3, TRPM2, RIPK1, TRPC6, TRPM1, MCOLN1, TRPC3, TRPC7, TRPV4, TRPM7, TRPM4, TRPM3, MLKL, and TRPV2 showed prevalent CNV deletions (**Supplementary Figure 1C**). TRPCR mutations were detected in 137 (34.34%) patients from 399 samples. **Supplementary Figure 1D** exhibited the landscape of the mutations, with TRPM5, TRPC3, and TRPC7 as the top three mutations.

Almost all TRPCR were downregulated, while MLKL, TRPC4AP, TRPM2, and TRPV4 were upregulated in the CRC tissues compared with normal tissues (P< 0.05, **Supplementary Figure 1E**). No significant differences were observed in MCOLN3, TRPC4, TRPM1, TRPM8, TRPV1, and TRPV5 (P > 0.05, **Supplementary Figure 1E**). Unfortunately, only two TRPCR, including TRPM5 and TRPV4 (HR > 1, P< 0.01), were associated with the OS of patients with CRC (**Supplementary Figure 1F**).

### Unsupervised clustering of 28 TRPCR and differences between two clusters

**Figure 2:**
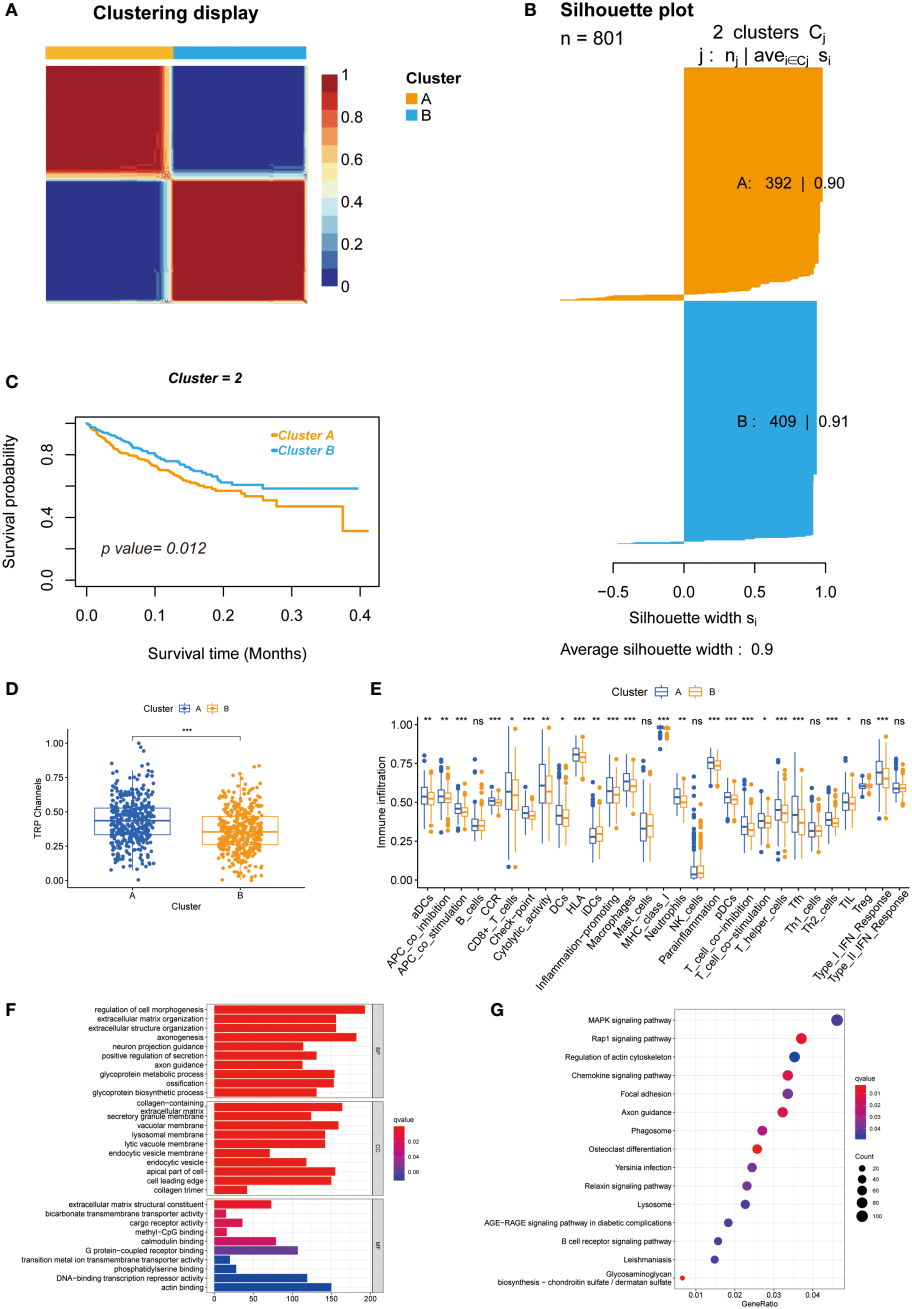
Unsupervised clustering of 28 TRPCR and differences between two clusters. **(A)** Heatmap representation of NMF clustering for TRPCR. **(B)** Kaplan-Meier curves of OS with two TRPC clusters. **(C)** Silhouette plot of the two clusters. **(D)** The TRPC activity between two clusters. **(E)** The ssGSEA scores of immune cells and immune-related functions with two clusters. **(F, G)** Functional annotation for TRPC clusters using GO and KEGG enrichment analysis. GO, Gene Ontology; KEGG, Kyoto Encyclopedia of Genes and Genomes; OS, overall survival; ssGSEA, single sample gene set enrichment analysis; TRPC, transient receptor potential channels; TRPCR, transient receptor potential channels regulators; ns, not significant; *P< 0.05; **P< 0.01; ***P< 0.001.

As shown in **Supplementary Figure 2**, the highest intra-group correlations and lowest inter-group correlations were observed when k = 2 in the TCGA and meta-GEO cohorts, indicating that patients can be divided into cluster A and cluster B based on 28 TRPC-associated DEGs (DEG_a_). **Figure 2A** exhibited two distinct patterns of CRC samples, which had two apparently different Kaplan–Meier survival curves (P< 0.05, **Figure 2B**). The silhouette plot of the two clusters is shown in **Figure 2C**. Interestingly, TRPC was more enriched in cluster A compared with cluster B (P< 0.05, **Figure 2D**). The single sample gene set enrichment analysis (ssGSEA) scores of aDCS, antigen-presenting cells (APC) co-inhibition, APC co-stimulation, chemokine receptor (CCR), CD8^+^ T-cells, immune checkpoint, cytolytic activity, dendritic cells (DCs), human leukocyte antigen (HLA), inflammation-promoting, macrophages, major histocompatibility complex (MHC) class I, neutrophils, parainflammation, plasmacytoid dendritic cell (pDCs), T cell co-inhibition, T cell co-stimulation, T helper (Th) cells, follicular helper T cell (Tfh), Th2 cells, tumor infiltrating lymphocyte (TIL), and Type I interferon (INF) response were significantly higher in cluster A than that in cluster B; while it was on the contrary in terms of iDCs (P< 0.05, **Figure 2E**). GO membrane-related pathways were enriched in cellular components (CC) (**Figure 2F**), and KEGG enrichment analysis showed that MAPK ranked first among the enriched signaling pathways (**Figure 2G**).

### Screening of characteristic predictors and prognostic value of TRPCRS

**Figure 3:**
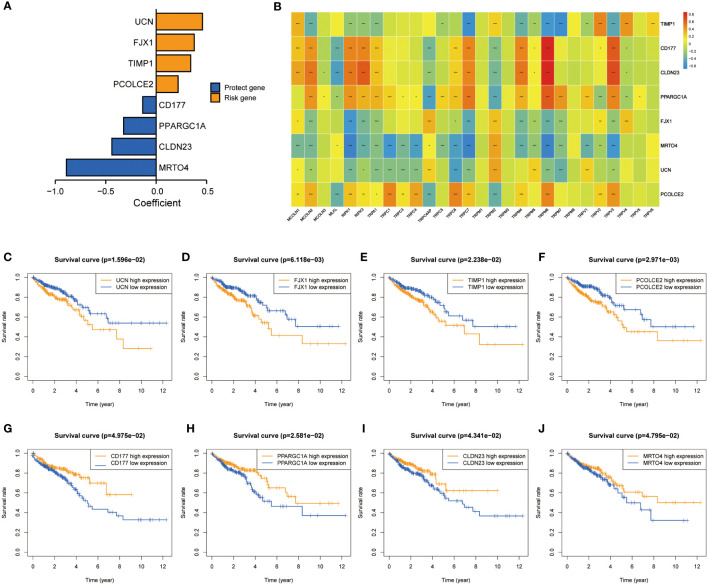
Screening of characteristic predictors and prognostic value of TRPCRS. **(A)** The coefficient value of the 8 TRPCGs associated with the TRPC score in CRC. **(B)** The correlation between 8 TRPCGs and 28 TRPCR. **(C-J)** Survival curve analysis of CRC patients based on the expression status of UCN, FJX1, TIMP1, PCOLCE2, CD177, PPARGC1A, CLDN23, MRTO4 genes. CRC, colorectal cancer; TRPCGs, transient receptor potential channels-related genes; TRPCR, transient receptor potential channels regulators; TRPCRS, transient receptor potential channels related signature; ns, not significant; *P< 0.05; **P< 0.01; ***P< 0.001.

A total of 5564 genes were identified as DEG_a_ (**Table S3**), 4605 as DEG_b_ (**Table S4**), and 1329 as prognostic-related genes (**Table S5**). Among these, 266 intersected genes were selected as candidate genes. **Supplementary Figure 3A** shows the coefficients of all 266 intersected genes TRPCG according to lambda.min criteria. Using the LASSO regression analysis, 8 gene signatures (UCN, FJX1, TIPM1, PCOLCE2, CD177, PPARGC1A, CLDN23, and MTOR4) were optimal with a minimum lambda (**Supplementary Figure 3B**). Among these 8 genes, 4 were risk factors (UCN, FJX1, TIPM1, and PCOLCE2), and 4 were protective factors (CD177, PPARGC1A, CLDN23, and MTOR4) (**Figure 3A**). The correlations between 8 TRPCG and 28 TRPCR are shown in **Figure 3B**. The apparent Kaplan–Meier survival curves between different groups stratified by the expression of 8 genes are shown in **Figures 3C–J** (P< 0.05). TRPC scores were calculated according to the following formula: TRPC score = [UCN expression×(0.4591)] + [FJX1 expression×(0.3770)] + [TIMP1 expression×(0.3425)] + [PCOLCE2 expression×(0.2178)] + [CD177 expression×(−0.1330)] + [PPARGC1A expression×(−0.3223)] + [CLDN23 expression×(−0.4393)] + [MRTO4 expression×(−0.8897)]. Interestingly, Gene set variation analysis (GSVA) showed that TRPC activity was positively correlated with the TRPC score (**Supplementary Figure 3C**).

### Prognostic analysis of TRPC scores

**Figure 4:**
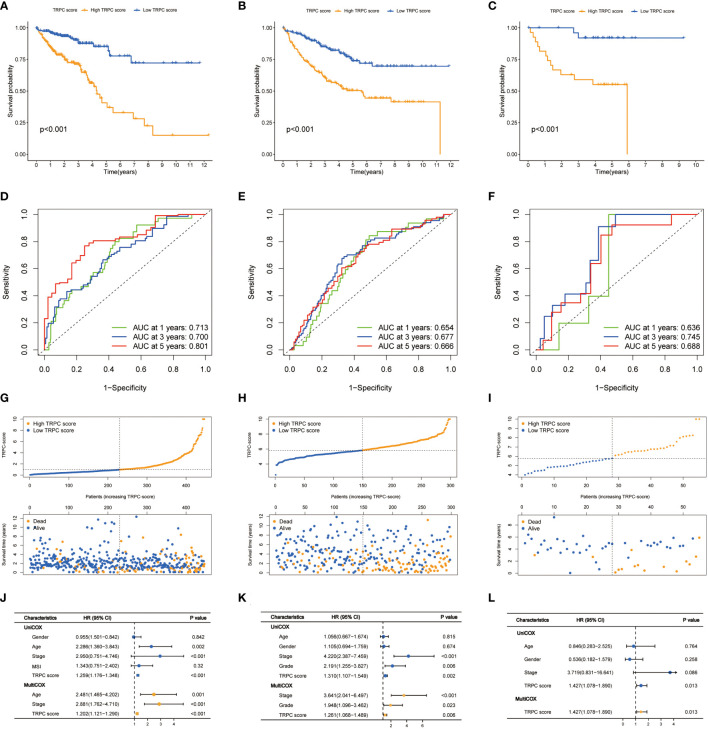
Prognostic analysis of TRPC scores. Kaplan–Meier curves **(A-C)**, time-dependent ROC curves **(D-F)**, risk plot distribution and survival status **(G-I)**, univariate and multivariate Cox regression analysis **(J-L)** of the TCGA cohort, the meta-GEO cohort, and the GSE17537 cohort. ROC, receiver operator characteristic; TRPC, transient receptor potential channels.

Considering the median score as the cut-off value, patients in the TCGA cohort were divided into low- and high-TRPC score subgroups. The Kaplan–Meier survival curve showed that the median OS was significantly shorter in the high-TRPC score subgroup than in the low-TRPC score subgroup (high vs. low, HR = 2.33, 95% confidence interval: 1.64–3.31, P< 0.001, **Figure 4A**). The area under the curve (AUC) at 1-year, 3-year, and 5-year were 0.713, 0.700, and 0.801, respectively (**Figure 4D**). The distinct distribution status of patients between the high- and low-TRPC score subgroups is shown in **Figure 4G**. Univariate Cox regression analysis showed that TRPC score was negatively associated with OS of patients with CRC. Additionally, multivariate Cox regression analysis showed that TRPC score was an independent risk factor for OS (both P< 0.05, **Figure 4J**). Similar findings were observed in the meta-GEO (**Figure 4B, E, H, K**) and GSE17537 cohorts (**Figures 4C, F, I, L**).

Subgroup analyses stratified by different characteristics were conducted to evaluate the correlations between the current TRPC score and other clinicopathological characteristics (**Supplementary Figure 4A**). No significant differences were observed in TRPC scores stratified by sex (female vs. male, P > 0.05, **Supplementary Figure 4B**) and age (≤65 vs. >65, P > 0.05, **Supplementary Figure 4C**). No significant differences were observed among all subgroups stratified by stage (P > 0.05). However, patients in stage IV had higher TRPC scores than those in other stage groups (all P< 0.05, **Supplementary Figure 4D**). Interestingly, patients with microsatellite stability (MSS) had lower TRPC scores than those with microsatellite instability-low (MSI-L) and microsatellite instability-high (MSI-H), but no differences were observed between MSI-L and MSI-H (P > 0.05, **Supplementary Figure 4E**).

### GO and KEGG analyses

GO analysis showed that replication-related biological processes (BP), mitochondria-related CC, and division-related molecular functions (MF) were enriched in the TCGA cohort (**Supplementary Figure 5A**). Enriched signaling pathways associated with CRC and mismatch repair were identified using KEGG analysis (**Supplementary Figure 5B**). Furthermore, membrane-related CC was enriched in the meta-GEO cohort (**Supplementary Figure 5C**), and PI3K-AKT and PPAR signaling pathways were identified using KEGG analysis (**Supplementary Figure 5D**).

### Immune landscapes and prediction of immunotherapeutic benefits

**Figure 5:**
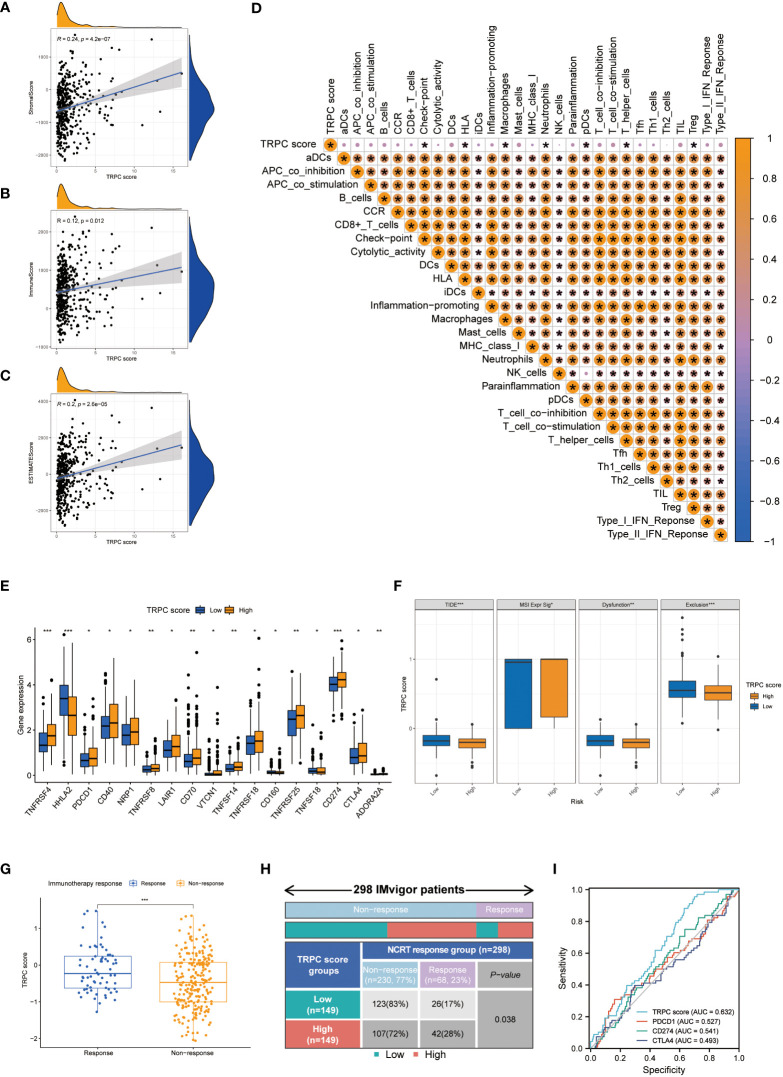
Immune landscapes and prediction of immunotherapeutic benefits. Scatter plot showed the association between TRPC score and stromal score **(A)**, immune score **(B)** and ESTIMATE score **(C, D)** Heat map of correlation between TRPC score and immune infiltration. **(E)** The association of the immune check-points between low-and high-TRPC score groups. **(F)** The differences of TIDE score, MSI expression signature score, dysfunction score and immune exclusion score between low- and high-TRPC score groups. The scatter diagram of the TRPC score between response and non-response group **(G)**, and fourfold table between TRPC score and immunotherapy response **(H)** in the IMvigor dataset. **(I)** ROC curves to predict the response of atezolizumab in IMvigor dataset by TRPC score, PDCD1, CD274, and CTLA4. **ESTIMATE:** estimation of stromal and immune cells in malignant tumour tissues using expression data; MSI, microsatellite instability; ROC, receiver operator characteristic; TIDE, tumor immune dysfunction and exclusion; TRPC, transient receptor potential channels; ns, not significant; *P< 0.05; **P< 0.01; ***P< 0.001.

The association between TRPC and estimation of stromal and immune cells in malignant tumour tissues using expression data (ESTIMATE) scores was investigated using the ESTIMATE algorithm, which showed that stromal, immune, and ESTIMATE scores were positively correlated with TRPC score (P< 0.05, **Figures 5A–C**). Further, neutrophils, and regulatory T cells (Treg) were negatively associated with TRPC score (P< 0.05, **Figure 5D**). However, immune checkpoint, HLA, macrophages, pDCs, and T helper_cells were positively associated with the TRPC score (P< 0.05, **Figure 5D**).

In addition, significant differences were detected between the low- and high-TRPC score subgroups in most immune checkpoints (P< 0.05, **Figure 5E**). Compared with the low-TRPC score group, the high-TRPC score group had lower TIDE, dysfunction, and exclusion scores, while higher MSI expression score (P< 0.05; **Figure 5F**).

The IMvigor 210 dataset (29), including clinical information and RNA-seq data of patients with metastatic uroepithelial carcinoma treated with atezolizumab (a PD-L1 inhibitor), was used as an external cohort to test the predictive value of TRPC score for immunotherapy efficacy. The results showed that TRPC score was significantly higher in the response group than that in the non-response group (P< 0.001, **Figure 5G**). According to the current TRPC score, the response rate in the low-TRPC score group was significantly lower than that in the high-TRPC score group (P< 0.05, **Figure 5H**). The AUC of the current TRPC score to predict the response of atezolizimab was 0.632, which was higher than that of PDCD1 (PD-1), CD274 (PD-L1), and CTLA4 (**Figure 5I**).

### Summary of CRC mutation of TRPC score groups

As shown in **Supplementary Figure 6A**, somatic mutations occurred in 160 (96.39%) of 166 samples with high TRPC score. The detailed mutations, including variant classification, single-nucleotide polymorphism (SNP) type, and single-nucleotide variant (SNV) class, were depicted in **Supplementary Figure 7A**. Further, somatic mutations occurred in 143 (97.28%) of 147 samples with low TRPC score (**Supplementary Figure 6B**), and the corresponding mutations were summarized in **Supplementary Figure 7B**. TMB was positively associated with TRPC score (R = 0.14, P< 0.05, **Supplementary Figure 6C**), and Kaplan–Meier survival curve showed that patients with low TMB had a worse OS than those with high TMB (P< 0.05, **Supplementary Figure 6D**). Significant survival differences were observed between patients with high and low TMB stratified by TRPC score (P< 0.05, **Supplementary Figures 6E, F**).

### Development of a nomogram

**Figure 6:**
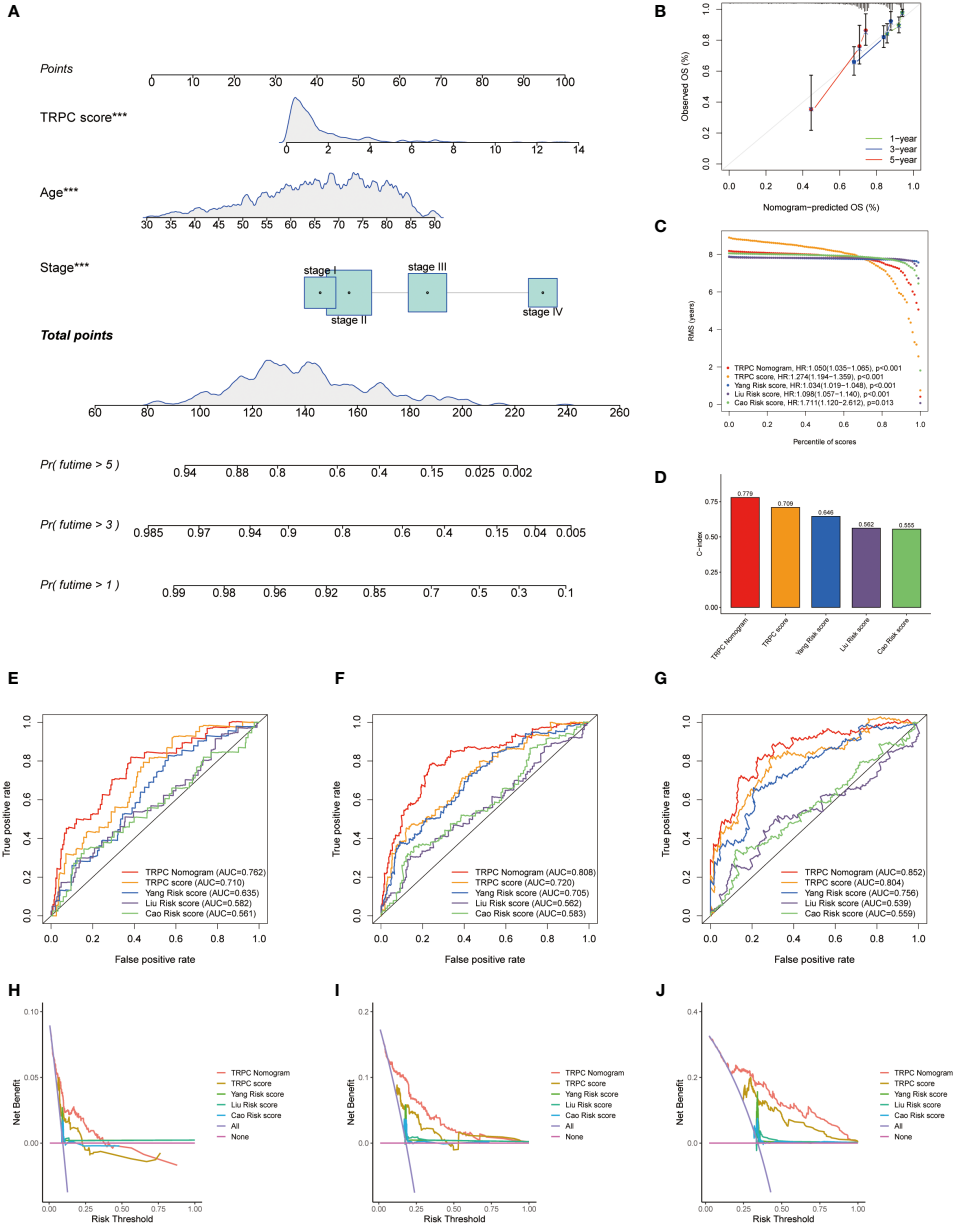
Development of a nomogram. **(A)** Nomogram to predict the survival of CRC patients based on the TRPC score. **(B)** Calibration plots of the nomogram for predicting the probability of OS in the 1-year, 3-year, and 5-years. Comparison of RMS **(C)** and C-index **(D)** among the TRPC nomogram, TRPC score, and published models of Yang, Liu, and Cao. The ROC curves **(E-G)** and decision curve analysis **(H-J)** at 1-, 3-, and 5-year OS for TRPC nomogram, TRPC score, and published models of Yang, Liu, and Cao. CRC, colorectal cancer; OS, overall survival; RMS, restricted mean survival; ROC, receiver operator characteristic; TRPC, transient receptor potential channels; ***P< 0.001.

A nomogram including age, stage, and TRPC score was developed to predict the OS of patients with CRC in the TCGA cohort (**Figure 6A**). Good calibrations were observed in the 1-year, 3-year, and 5-year predicted vs. observed survival rates (**Figure 6B**). The RMS of the TRPC nomogram was higher than that of the TRPC score and published models of Yang (32), Liu (33), and Cao (34) (P< 0.05, **Figure 6C**). The C-index of the TRPC nomogram was 0.779, which was higher than that of the TRPC score and published models of Yang (32), Liu (33), and Cao (34) (**Figure 6D**). ROC curves revealed that the TRPC nomogram predicted the 1-year, 3-year, and 5-year OS more efficiently than the TRPC score and published models of Yang (32), Liu (33), and Cao (34) (**Figures 6E–G**). As shown in **Figures 6H–J**, DCA curves showed that the TRPC nomogram had better 1-year, 3-year, and 5-year OS net benefit than the TRPC score and published models of Yang (32), Liu (33), and Cao (34).

### Correlation between TRPC score and response to neoadjuvant chemoradiotherapy in the GSE45404 cohort

**Figure 7:**
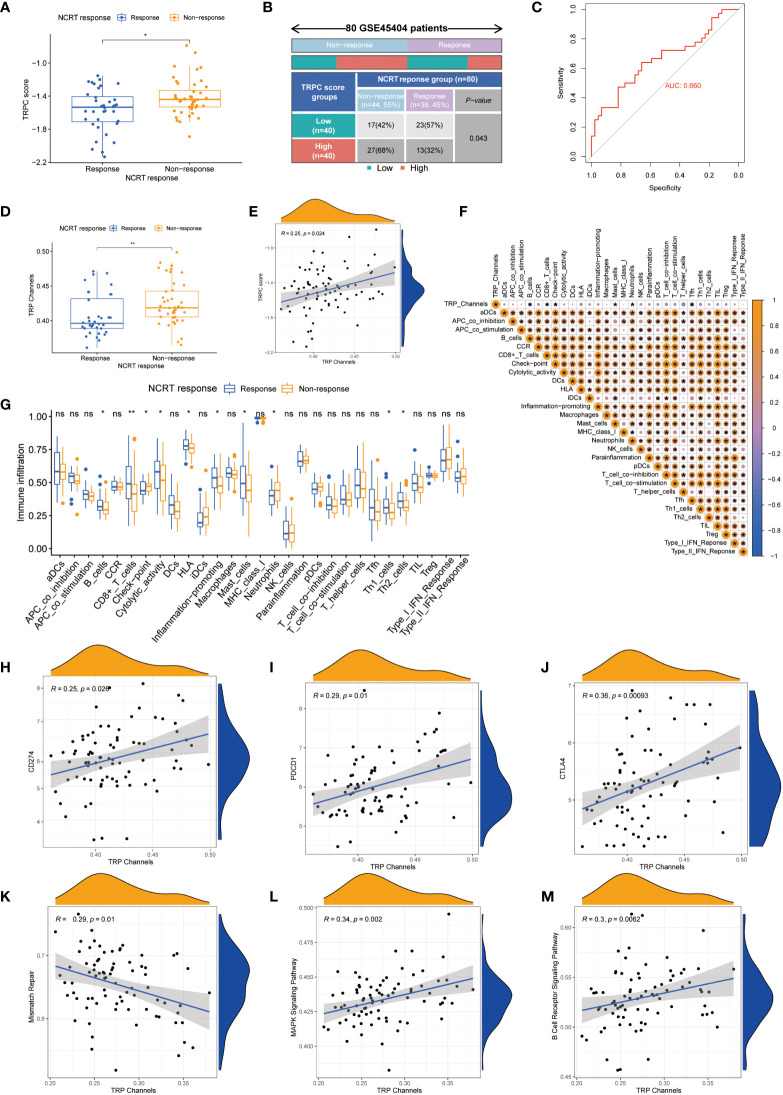
Correlation between TRPC score and response to NCRT in the GSE45404 cohort. The scatter diagram of the TRPC score between response and non-response group **(A)**, and fourfold table between TRPC score and NCRT response **(B)**. **(C)** ROC curve of TRPC score to predict NCRT response. **(D)** The distribution of the TRPC activity between response and non-response group. **(E)** Scatter plot showed the association between the TRPC activity and TRPC score. **(F)** Heat map of correlation between the TRPC activity and immune infiltration. **(G)** ssGSEA scores of immune cells and immune-related functions between response and non-response group. **(H-J)** Scatter plot showed the association between immune checkpoints (e.g., PD-1, PD-L1, and CTLA4) and the TRPC activity. **(K-M)** Scatter plot showed the association between signaling pathway (e.g., mismatch repair, MAPK signaling pathway, and B cell receptor signaling pathway) and the TRPC activity. NCRT, neoadjuvant chemoradiotherapy; ROC, receiver operator characteristic; ssGSEA, single sample gene set enrichment analysis; TRPC, transient receptor potential channels; ns, not significant; *P< 0.05; **P< 0.01.

**Figure 7A** shows that TRPC score was significantly lower in the response group than that in the non-response group (P< 0.05). According to the current TRPC score, the response rate in the low-TRPC score group was significantly higher than that in the high-TRPC score group (P< 0.05, **Figure 7B**). Further analysis showed that the current TRPC score had a promising predictive power of neoadjuvant chemoradiotherapy (NCRT) response (**Figure 7C**). GSVA showed that TRPC activity was significantly lower in the response group than that in the non-response group (P< 0.05, **Figure 7D**), and positively correlated with the TRPC score (P< 0.05, **Figure 7E**). B-cells, CD8^+^ T-cells, mast cells, and Tfh were negatively correlated, whereas immune checkpoint and neutrophils were positively correlated with TRPC activity (**Figure 7F**). Significantly increased proportions of B-cells, CD8^+^ T-cells, cytolytic activity, HLA, inflammation-promoting, mast cells, Th1 cells, and Th2 cells were detected in the response group. While, immune checkpoint and neutrophils were significantly increased in the non-response group (P< 0.001, **Figure 7G**). Further analysis showed that immune checkpoints, including PDCD1 (PD-1), CD274 (PD-L1), and CTLA4, and signaling pathway activities, including mismatch repair, MAPK, and B-cell receptors, were associated with TRPC activity (P< 0.05, **Figures 7H–M**).

### Correlation between TRPC score and prognosis of patients who received NCRT in the GSE87211 cohort

**Figure 8:**
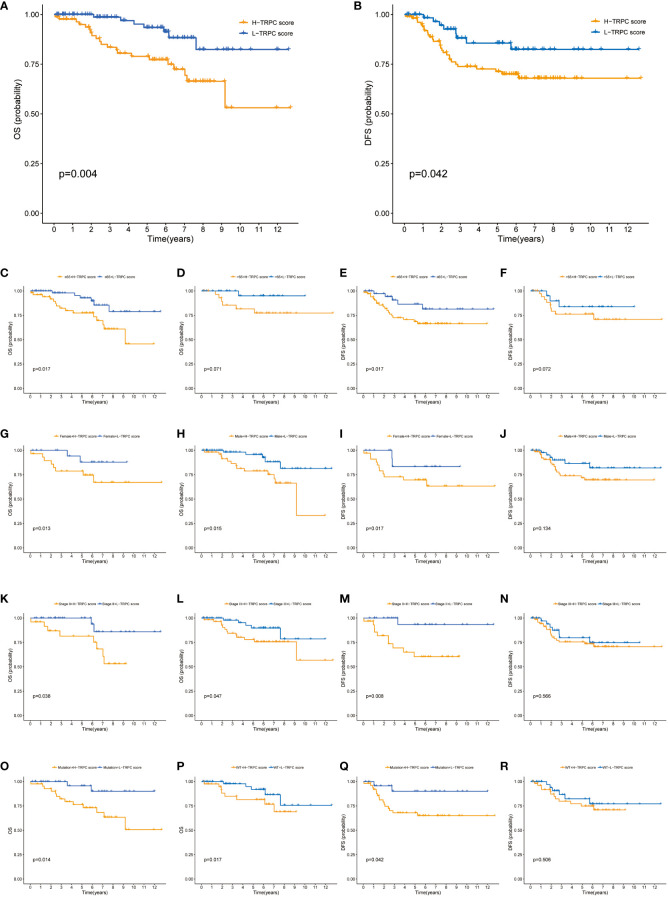
Correlation between TRPC score and prognosis of patients who received NCRT in the GSE87211 cohort. **(A)** The Kaplan-Meier curves of OS for patients in the high- and low-TRPC score groups. The Kaplan-Meier curves of OS for supplement clinicopathological characteristics, including age **(C, D)**, gender **(G, H)**, stage **(K, L)**, and KRAS mutation status **(O, P)** in the high- and low-TRPC score groups. **(B)** The Kaplan-Meier curves of DFS for patients in the high- and low-TRPC score groups. The Kaplan-Meier curves of DFS for supplement clinicopathological characteristics, including age **(E, F)**, gender **(I, J)**, stage **(M, N)**, and KRAS mutation status **(Q, R)** in the high- and low-TRPC score groups. NCRT, neoadjuvant chemoradiotherapy; OS, overall survival; TRPC, transient receptor potential channels.

The GSE87211 cohort tested the prognosis prediction capacity of TRPC score in patients who received NCRT. Results of this cohort showed that patients with low-TRPC scores had a longer OS and disease-free survival (DFS) than those with high-TRPC scores (P< 0.05, **Figures 8A, B**). Significant survival benefits in OS were observed in almost all subgroups (age≤65, **Figure 8C**; female, **Figure 8G**; male, **Figure 8H**; stage II, **Figure 8K**; stage III, **Figure 8L**; mutation, **Figure 8O**; wild type, **Figure 8P**; P< 0.05). However, there was no significant difference in age> 65 subgroup (**Figure 8D**, P>0.05). Similar findings were observed in DFS (age, **Figures 8E, F**; gender, **Figures 8I, J**; stage, **Figures 8M, N**; KRAS status, **Figures 8Q, R**).

### Validation of TRPC score in the Fujian Cancer Hospital cohort

**Figure 9:**
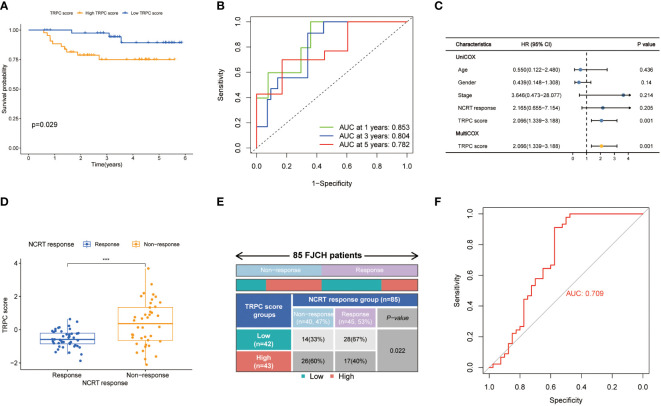
Validation of TRPC score in the Fujian Cancer Hospital cohort. Kaplan-Meier curve **(A)**, 1-year, 2-year, and 3-year ROC curves **(B)** and univariate and multivariate Cox regression analysis **(C)** according to the TRPC score. The scatter diagram of the TRPC score between response and non-response group **(D)**, and fourfold table between TRPC score and NCRT response **(E)**. **(F)** ROC curve of TRPC score to predict NCRT response. NCRT, neoadjuvant chemoradiotherapy; ROC, receiver operator characteristic; TRPC, transient receptor potential channels; ***P< 0.001.

A total of 85 samples were used from FJCH to verify the clinical value of the current TRPC score. Kaplan–Meier survival curve showed distinct survival differences between groups of high- and low-TRPC scores, according to the current TRPC scores (P< 0.05, **Figure 9A**). It also exhibited excellent prognosis prediction with a 5-year AUC of 0.782 (**Figure 9B**). Multivariate regression analysis showed that TRPC score was the only independent risk factor of OS (P< 0.05, **Figure 9C**). Furthermore, the TRPC score was significantly lower in the response group to neoadjuvant treatment than that in the non-response group (P< 0.05, **Figure 9D**). The response rate in the low-TRPC score group was significantly higher than that in the high-TRPC score group (P< 0.05, **Figure 9E**). The current TRPC score had inspiring predictive ability of response to neoadjuvant treatment with AUC of 0.709 (**Figure 9F**).

## Discussion

Ion channels, particularly TRPC, are crucial in cancer pathophysiology (7). TRPC are often dysregulated in CRC, resulting in alterations in cancer hallmark functions (16, 17). To the best of our knowledge, the present study is the first to systematically evaluate TRPC in CRC. A risk score incorporating 8 TRPCG was established to predict the OS of patients with CRC using the TCGA cohort, and was validated using the GSE38832, GSE17536, and GSE17537 datasets. Furthermore, the TRPC score was associated with other clinicopathological characteristics of patients with CRC and tumor immunity.

Since 1969, many TRPC family members have been identified, regulating numerous cellular, physiological, and pathophysiological functions in tumors (6–8, 13). Previous studies revealed that TRPC1 (35), TRPV6 (36, 37), and TRPM8 (38) were upregulated in CRC tissues compared with normal mucosa, whereas TRPV3 (39), TRPV4 (40), TRPV5 (39), TRPM6 (41), and TRPC6 (39) were downregulated in CRC tissues. However, no systematic study on the role of TRPC in the prognosis of CRC has been reported yet. In the current study, 28 TRPCR were identified from TCGA, showing that almost all 28 TRPCR were dysregulated in CRC tissues than that in normal tissues. However, TRPM5, TRPV4, and TRPC1 were associated with OS of patients with CRC (P< 0.05). Two clusters with distinct prognoses were identified using NMF (P< 0.05), and TRPC were enriched between clusters A and B. Further, immunophenotypes differed significantly between the two clusters, indicating that TRPC might regulate the immune system as reported previously (20, 41).

Considering that studies on TRPCR are scarce, we identified TRPCG, which were not only DEGs between two clusters, but also between tumor and normal samples. In addition, TRPCR were associated with prognosis, and all candidate genes strongly associated with TRPCR. The current TRPC score exhibited excellent prognosis predictive ability in the TCGA, meta-GEO, GSE17537, GSE87211, and FJCH cohorts, and was identified as an independent risk factor of OS (P< 0.05). Furthermore, a nomogram based on the current TRPC score showed higher C-index and AUC of prognosis prediction compared with published risk scores of previous studies by Yang et al. (32), Liu et al. (33), and Cao et al. (34), and improved net benefits. Moreover, the current TRPC score correlated with clinicopathological characteristics, including TNM stages and microsatellite status.

Among the 8 TRPCG, UCN, FJX1, TIPM1, and PCOLCE2 were negatively associated with OS of CRC patients (HR > 1), while CD177, PPARGC1A, CLDN23, and MTOR4 were positively associated with OS of CRC patients (HR< 1). Song et al. (42) reported that TIMP1 expression was significantly associated with regional lymph node and distant metastasis, and was an independent prognostic indicator of colon cancer progression and metastasis through FAk-PI3K/AKT and MAPK signaling pathways (42, 43). FJX1 was reportedly upregulated in the epithelium of CRC, and contributed to poor prognosis in patients with CRC *via* angiogenesis (44). CLDN23 and PPARGC1A were significantly downregulated in CRC tissues, and their reduced levels were associated with shorter OS in patients with CRC (45–47). PPARGC1A reduced the risk of CRC disease and progression through mitochondrial biogenesis, antioxidant system, reactive oxygen species, lipid synthesis, and glycolysis pathway (46). CD177 is mainly expressed by neutrophils, and CRC patients with high density CD177+ neutrophils showed longer OS and DFS (48). Although UCN, PCOLCE2, and MRTO4 are potential prognostic markers of CRC, their action mechanisms remain unclear. In summary, several candidate genes were first considered as prognostic biomarkers for CRC, however, they require further validation.

With promising results of clinical trial of pembrolizumab (49), ICI monotherapy or combination therapy has been well examined in CRC management (50, 51). In recent years, deficient mismatch repair (dMMR) showed the objective response rate of 20–40%, regardless of stages (50, 51). However, the incidence of patients with dMMR account for approximately 15% of CRC (52), and selected patients with proficient mismatch repair MSS precisely could benefit from ICIs (50, 53, 54). In the present study, TRPCR-based clusters were highly correlated with TIME. In addition, the current TRPCRS was correlated with immune checkpoints, including PD-1, PD-L1, and CTLA4. Additionally, the results showed that patients with high-TRPC scores had lower TIDE but higher MSI expression than those with low-TRPC scores (P< 0.05), suggesting that the former could benefit from ICIs. These findings were validated by an external IMvigor 210 cohort (29), in which all patients received atezolizumab. These findings demonstrated that the novel TRPC score was considered as a promising biomarker for TIME and an alternative index for ICIs. Nonetheless, the correlation between TRPC and immunotherapy is still inclusive, which needs further validation, and the underlying mechanism needs to be further explored.

Although neoadjuvant treatment followed by radical surgery is the standard treatment for locally advanced rectal cancer (LARC) (18), the pathological complete response rate and survival benefit remain unsatisfactory and questionable (5). Hence, a widely recognized biomarker is the key to selecting a potential beneficiary. In the present study, the current TRPCRS was associated with neoadjuvant treatment response in the GSE45404 cohort, and patients with low-TRPC scores were more sensitive to neoadjuvant treatment than those with high-TRPC scores and higher TRPC activity. In addition, the novel TRPCRS was associated with DFS in the GSE87211 cohort. Consequently, the current TRPCRS could be considered as an index of response to neoadjuvant treatment for patients with LARC, and neoadjuvant chemoradiotherapy should be strongly recommended for patients with low-TRPC scores.

More findings were recorded for patients with high-TRPC scores but tolerant to conventional NCRT in the present study. Initially, neutrophils were significantly enriched in the non-response group than in the response group (P< 0.05). Evidence showed that enrichment of neutrophils is one of the important characteristics of neutrophil extracellular trap (NET) (55), which might be a partial reason for chemoradiotherapy resistance, as previously reported (56–58). Previous studies showed that NET formation was positively regulated by MAPK signaling pathway (59, 60); however, the related mechanism remains unclear. In the present study, MAPK signaling pathway was positively correlated with TRPC activity (P< 0.05), which was positively correlated with TRPC score (P< 0.05). Hence, neutrophil enrichment mediated by activation of MAPK signaling pathway might be a potential mechanism for NCRT resistance, in which TRPC might play an important role. In future, it is a big “if” that combination with MAPK inhibitor and conventional chemoradiotherapy could improve the treatment response. Moreover, considering that patients with high-TRPC scores were more sensitive to ICIs than those with low-TRPC scores, neoadjuvant ICIs might be an alternative for those with high-TRPC scores and resistant to conventional chemoradiotherapy.

The current TRPCRS has several limitations. (1) Data from TCGA training cohort, meta-GEO, and FJCH validation cohorts were retrospectively collected. Therefore, the TRPC score should be validated by prospective cohorts. (2) A model consisting of TRPC score to predict the prognosis might have an intrinsic disadvantage, regardless of the importance of TRPCGs. (3) All eight genes were TRPC-related genes but not TRPCR. (4) Since all patients from FJCH had dMMR, corresponding analysis could not be conducted. (5) Mechanisms, such as MAPK signaling pathway, require *in vitro* and *in vivo* validation. (6) The clinical value of the current TRPCRS in ICIs and neoadjuvant treatment management needs further validation.

## Conclusion

In conclusion, a novel risk score was developed using eight TRPCG with excellent discrimination and calibration for CRC prognosis. The current TRPCRS could be considered as a promising biomarker for ICIs and neoadjuvant treatment in CRC management. NCRT is recommended for patients with LARC with low-TRPC scores. In addition, combination of MAPK inhibitors and neoadjuvant immunotherapy could be an alternative treatment for patients with high-TRPC scores. Hence, TRPC might participate in the treatment response and TIME remodeling in CRC management, but it requires further *in vitro* and *in vivo* validation.

## Data availability statement

The datasets presented in this study can be found in online repositories. The names of the repository/repositories and accession number(s) can be found in the article/**Supplementary Materials**.

## Ethics statement

The studies involving human participants were reviewed and approved by The Ethics Committee of Fujian Cancer Hospital. The patients/participants provided their written informed consent to participate in this study.

## Author contributions

LW, XtC, HjZ, LH, JcW, LdS, GC, and JxW contributed to conception and design. XtC and HjZ conducted data collection and analyzed the data. LW, LH, and JcW interpreted the data. LW, XtC, and LS drafted the manuscript. HjZ and JcW conducted the qRT-PCR experiment and proofread the experimental data. LdS, GC, and JxW contributed critical revision of the manuscript. All authors read and approved the final manuscript.

## Funding

This research was funded by the Science and Technology Program of Fujian Province, China (No.2019L3018 and 2019YZ016006), the Fujian Province Natural Science Foundation (2021J01438), the Fujian Provincial Clinical Research Center for Cancer Radiotherapy and Immunotherapy (2020Y2012), Fujian Provincial Health and Family Planning Research Talent Training Program (2020GGB009), the National Clinical Key Specialty Construction Program, and Fujian Province Gastrointestinal, Respiratory and Genitourinary Malignant Tumor Radiotherapy Radiation and Treatment Clinical Medical Research Center (2021Y2014).

## Acknowledgments

We thank the TCGA and GEO database for providing valuable and public datasets. The authors wish to thank Bullet Edits Limited for the linguistic editing and proofreading of the manuscript.

## Conflict of interest

The authors declare that the research was conducted in the absence of any commercial or financial relationships that could be construed as a potential conflict of interest.

The reviewer S.Y declared a shared parent affiliation with the authors H.Z, J.W, G.C to the handling editor at the time of review.

## Publisher’s note

All claims expressed in this article are solely those of the authors and do not necessarily represent those of their affiliated organizations, or those of the publisher, the editors and the reviewers. Any product that may be evaluated in this article, or claim that may be made by its manufacturer, is not guaranteed or endorsed by the publisher.
