# Supplementary material for: Comprehensive analysis of transient receptor potential channels-related signature for prognosis, tumor immune microenvironment, and treatment response of colorectal cancer
Source: Front Immunol. 2022 Oct 18;13:1014834. doi: 10.3389/fimmu.2022.1014834 (PMC9642045; doi:10.3389/fimmu.2022.1014834)
Supplement: Supplementary file 1 [file DataSheet_1.docx]

Supplementary Material

# Supplementary Data

## Original data

Because the original data is so large, we uploaded it to the following url: <https://www.jianguoyun.com/p/DTz6z3EQ-IbfChji1ssEIAA>

# Supplementary Figures and Tables

## Supplementary Figures

**
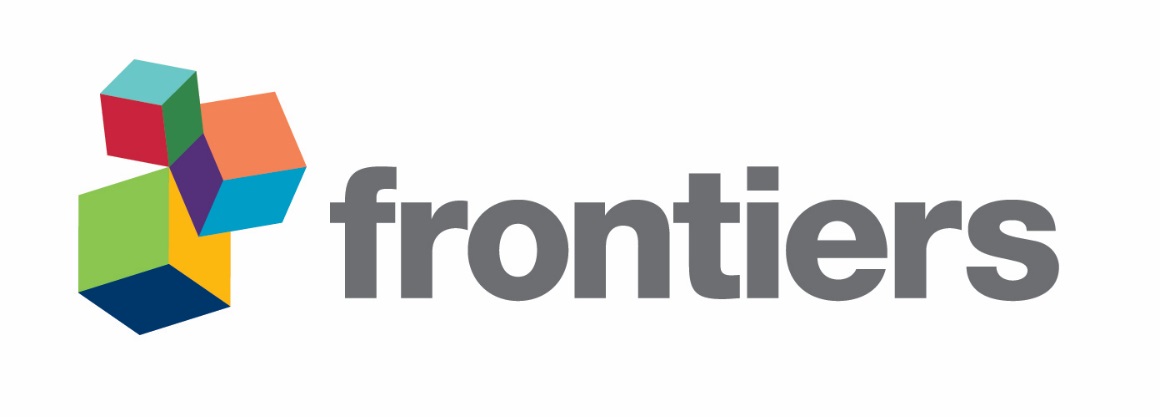
**


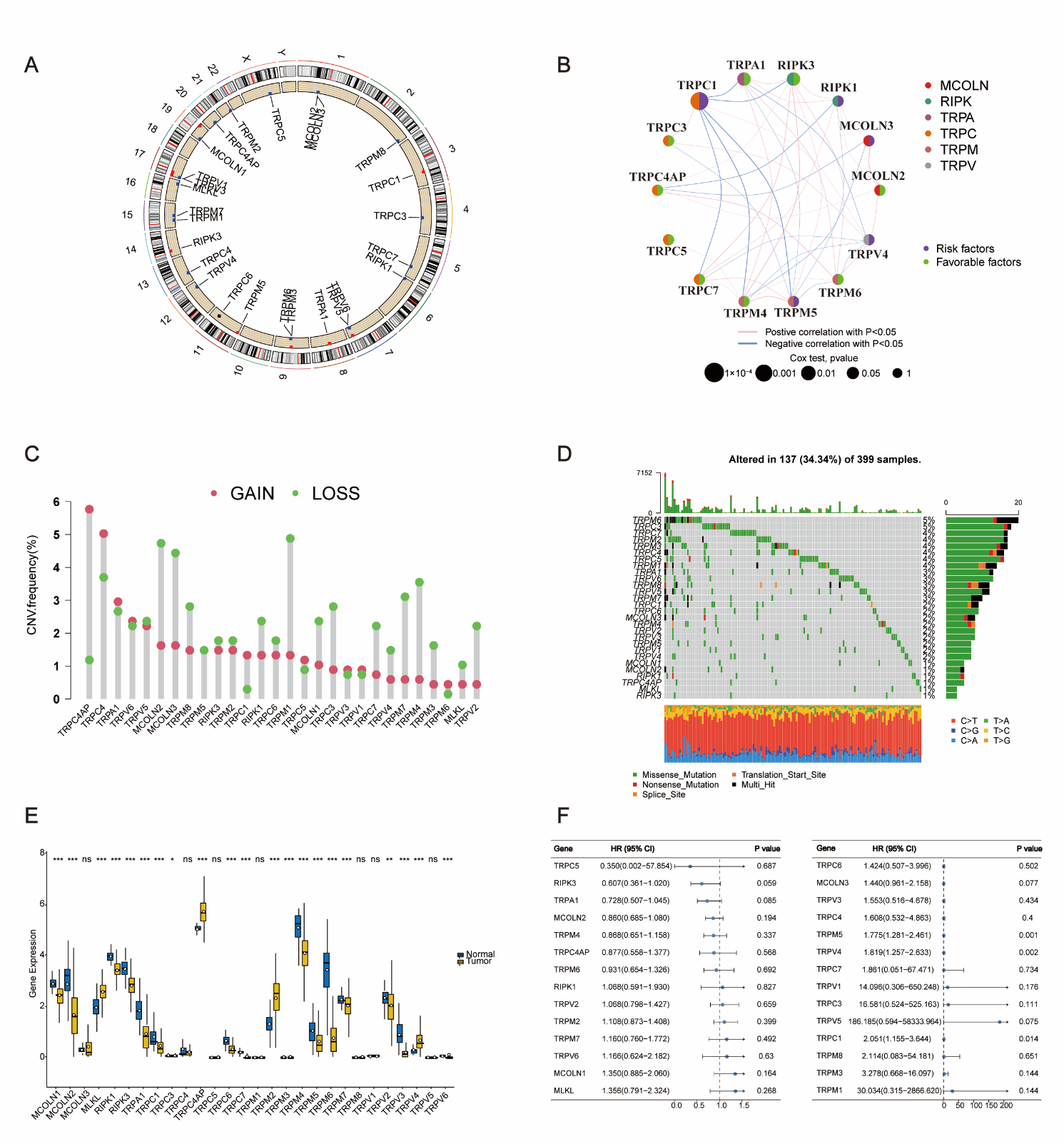
 **Supplementary Figure 1. Genetic alteration landscape of TRPCR in CRC.** **(A)** The landscape of genetic alterations of TRPCR. **(B)** The interaction of expression on 28 TRPCR. **(C)** The CNV mutation frequency of 28 TRPCR. **(D)** The landscape of mutation profiles of 28 TRPCR. **(E)** The difference of mRNA expression levels of 28 TRPCR between normal and CRC samples. **(F)** The results of the univariate Cox regression analysis between the expression of TRPCR and OS. **CNV:** copy number variations; **CRC:** colorectal cancer; **OS:** overall survival; **TRPCR:** transient receptor potential channels regulators.


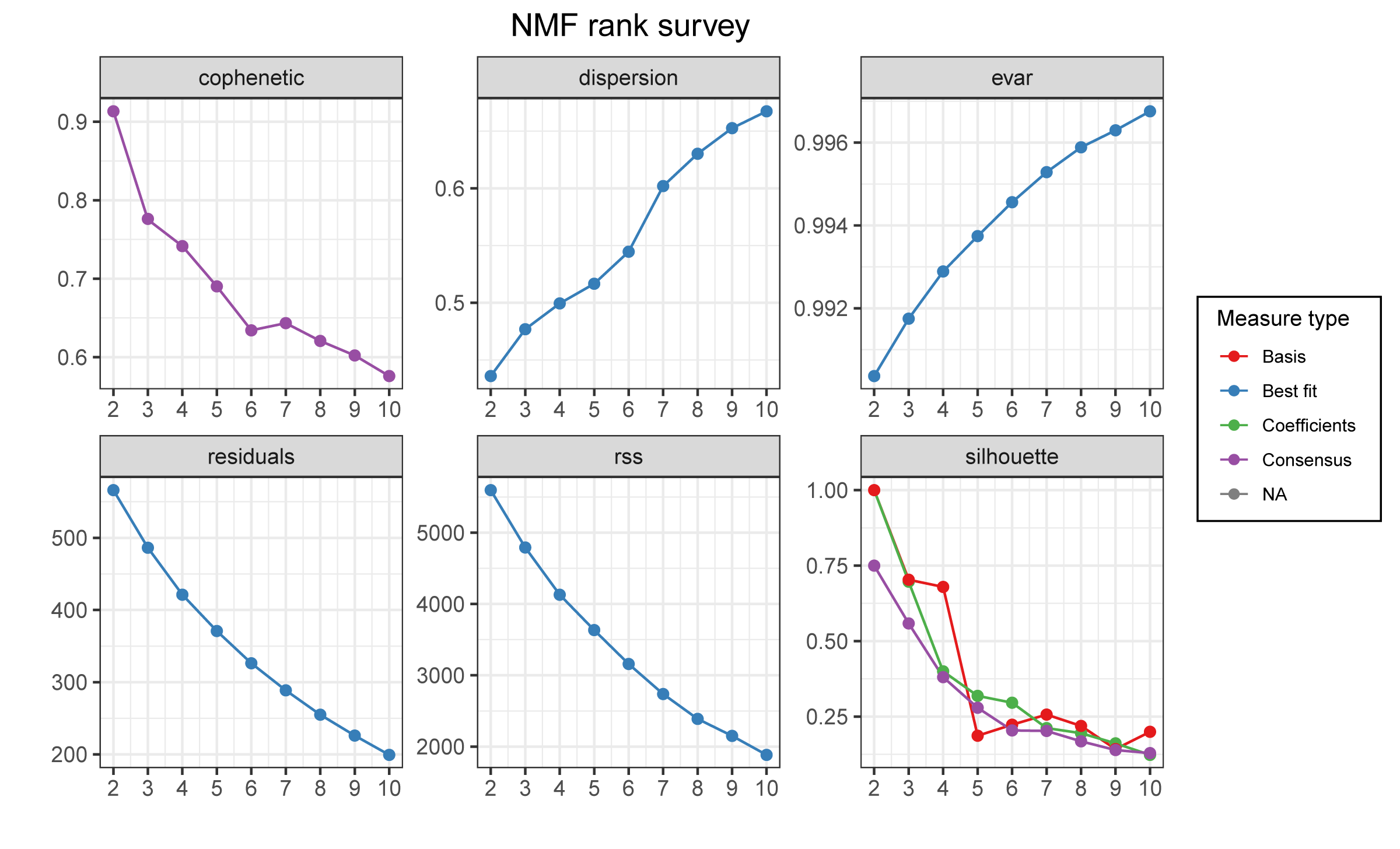


**Supplementary Figure 2.** **The relationship between cophenetic, dispersion, residuals, and silhouette coefficients with respect to the number of clusters.**

**
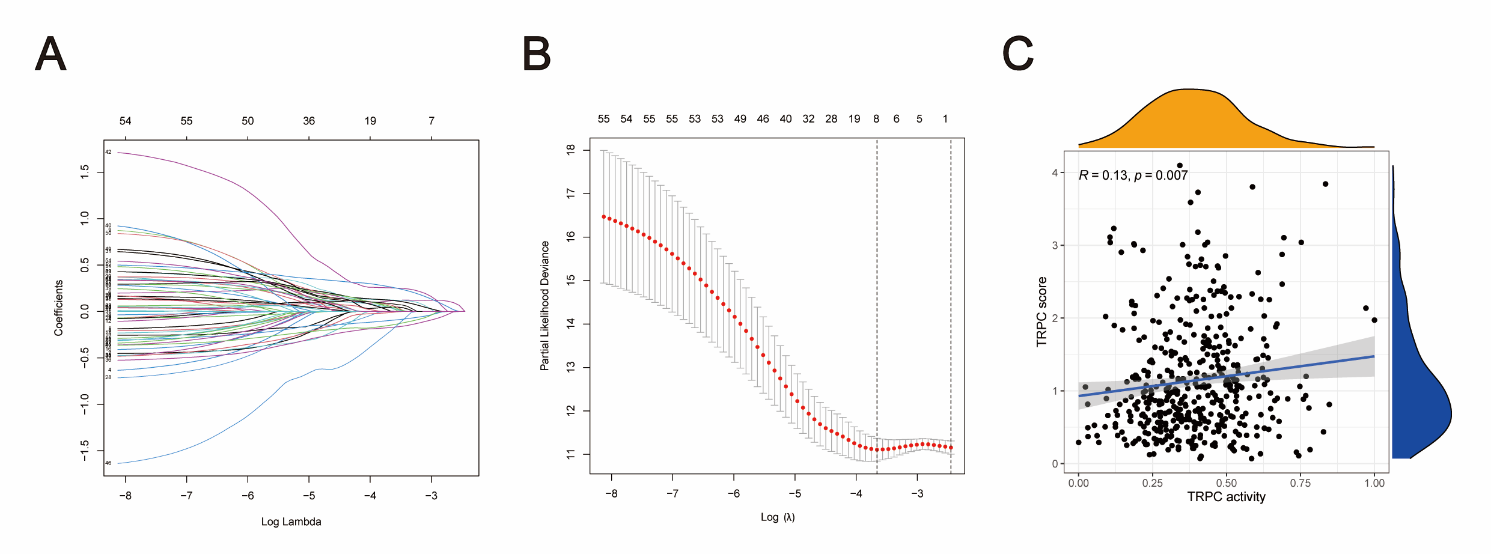
**

**Supplementary Figure 3.** **TRPC score construction and correlation analysis between TRPC score and TRPC activity. (A)** LASSO coefficient profiles of the expression of the candidate TRPCGs. **(B)** Selection of the penalty parameter (Lambda) in the LASSO model. **(C)** Scatter plot showed the association between the TRPC activity and TRPC score. **LASSO:** least absolute shrinkage and selection operator; **TRPC:** transient receptor potential channels; **TRPCGs:** transient receptor potential channels-related genes.


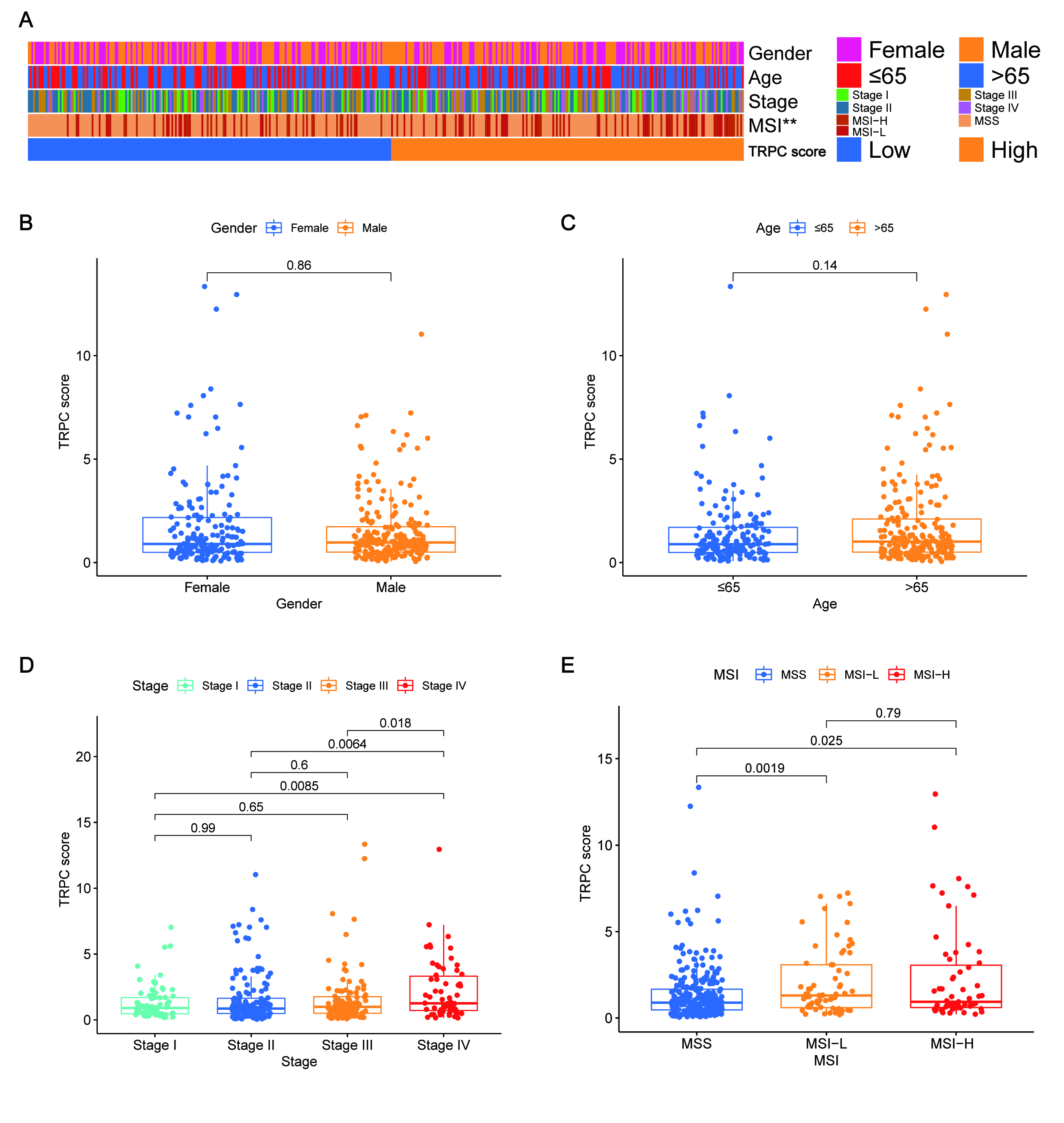


**Supplementary Figure 4.** **Clinicopathological characteristics evaluation by TRPC score.** Strip chart **(A)** along with heatmap and boxplot showing the distribution of TRPC score between the groups of gender **(B)**, age **(C)**, clinical stage **(D)** and MSI status **(E)**. **MSI:** microsatellite instability; **TRPC:** transient receptor potential channels.


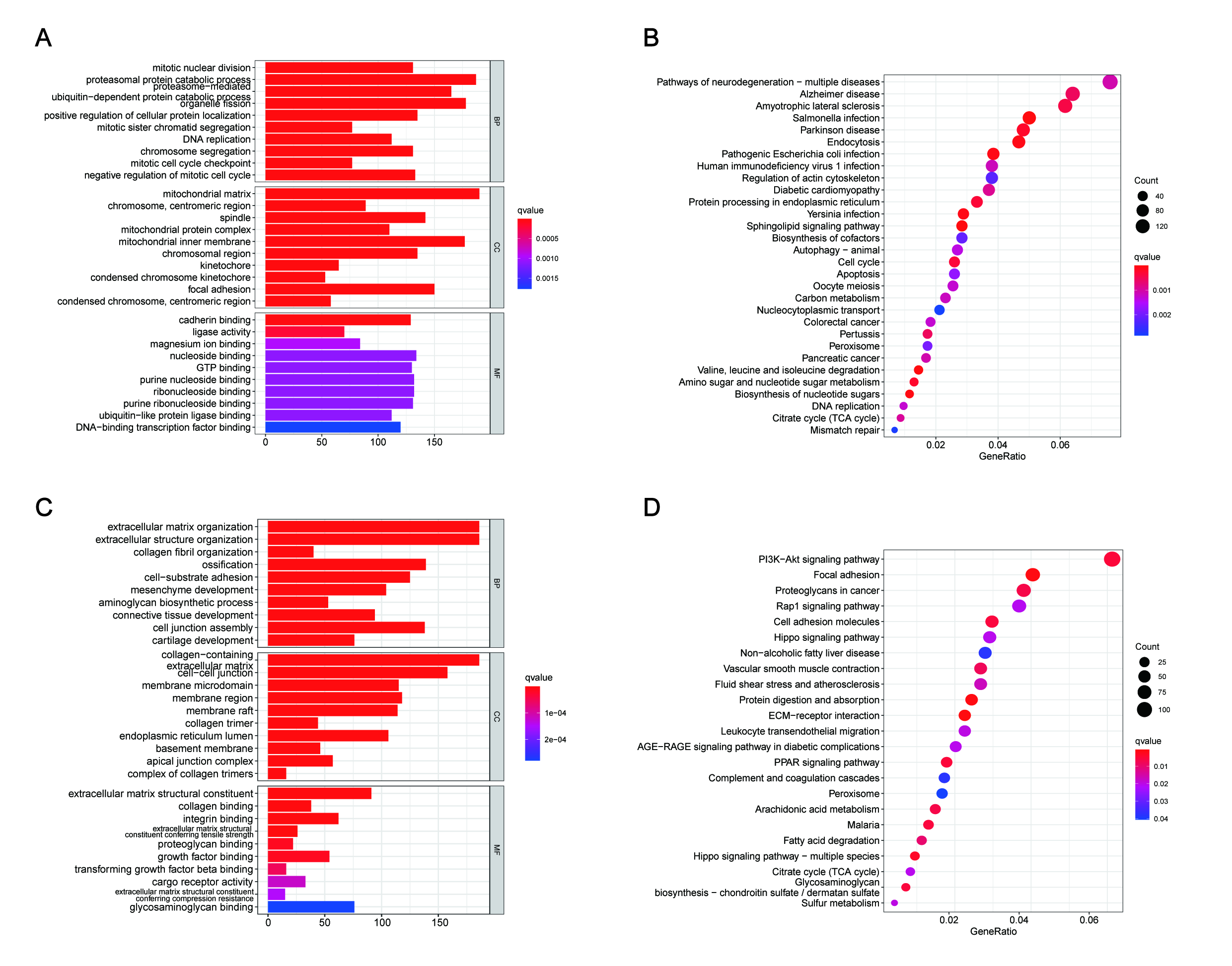


**Supplementary Figure 5. Representative results of GO and KEGG analyses.** The most significant GO enrichment and KEGG pathways in the TCGA cohort **(A, B)** and meta-GEO **(C, D)** are displayed. **GO:** Gene Ontology; **KEGG:** Kyoto Encyclopedia of Genes and Genomes.


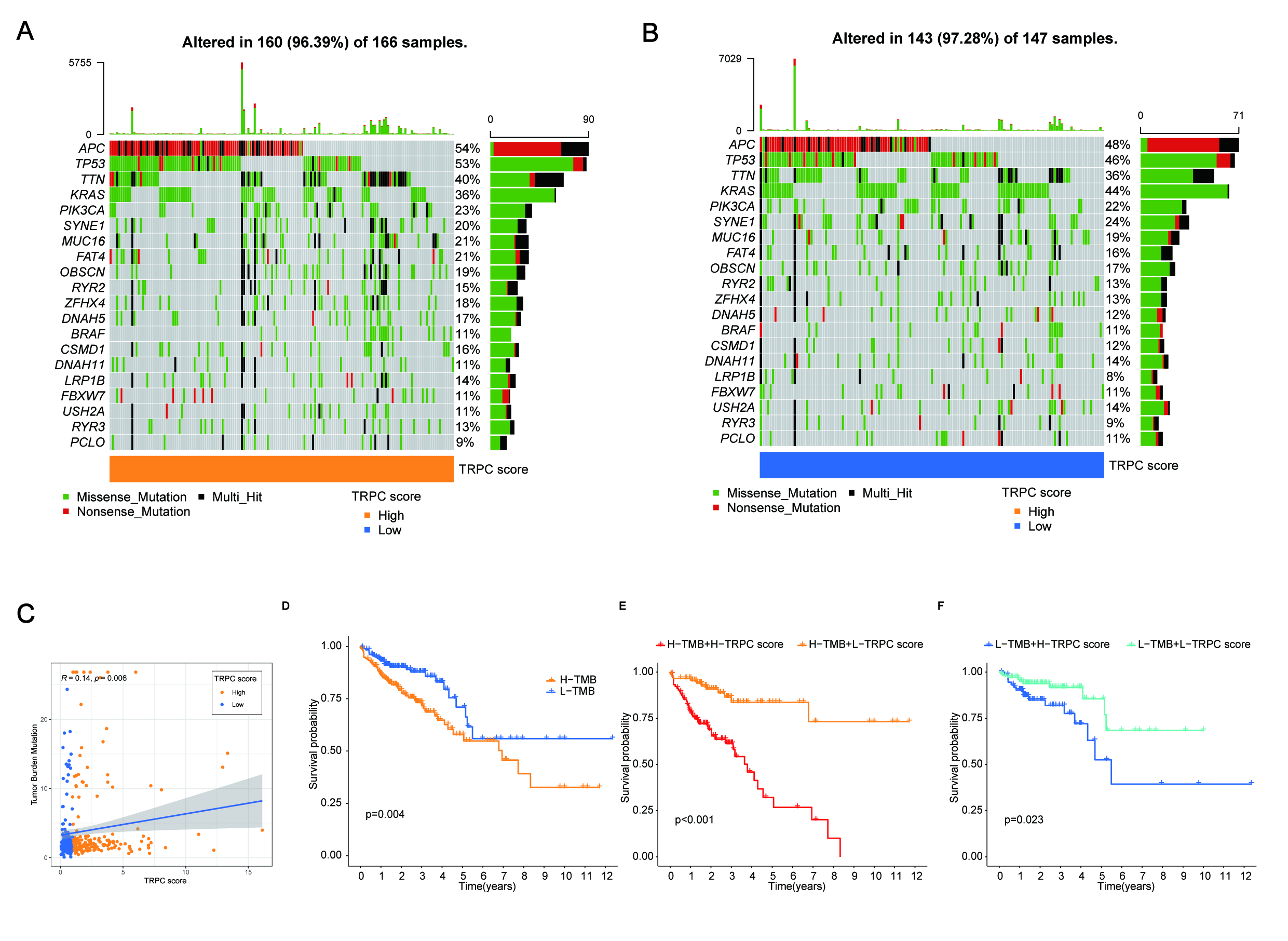
 **Supplementary Figure 6.** **CRC mutation information of TRPC score groups. (A-B)** Waterfall plot showing the landscape of mutation profiles in high- and low-TRPC score groups. **(C)**The association between TMB and low- and high-TRPC score. Kaplan–Meier curves of TMB **(D)**, high-TMB **(E)** and low-TMB **(F)** with low- and high-TRPC score. **CRC:** colorectal cancer; **TRPC:** transient receptor potential channels; **TMB:** tumor mutation burden.


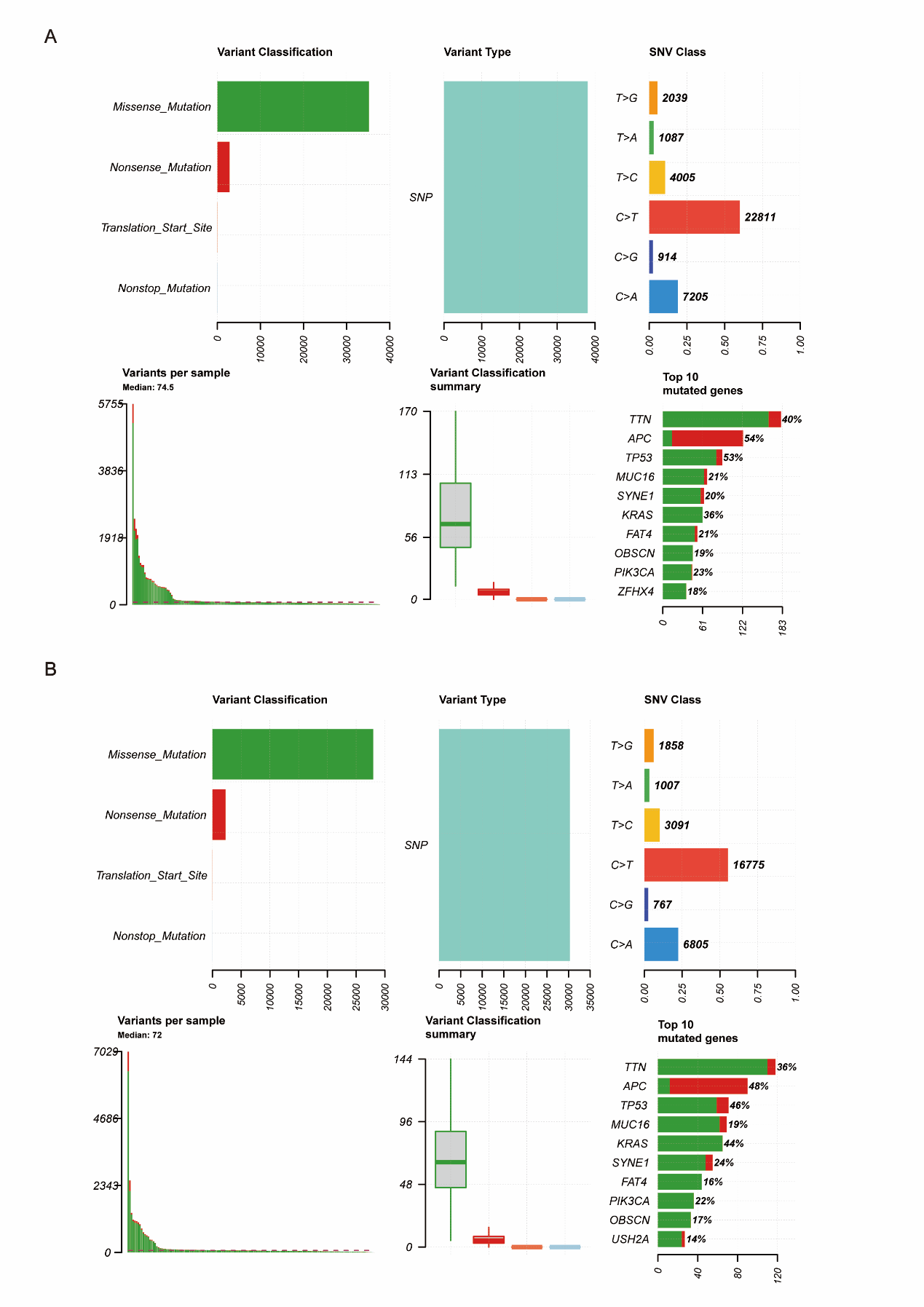


**Supplementary Figure 7. Summary of the of CRC mutation information of TRPC score groups.** Classification of mutation types according to different categories of specific samples in the low-TRPC score **(A)** and high-TRPC score **(B)** groups. **CRC:** colorectal cancer; **TRPC:** transient receptor potential channels.

## Supplementary Tables

**Supplementary Table 1.**  28 transient receptor potential channel-related genes.

**Supplementary Table 2.** The primers sequence used in this study.

**Supplementary Table 3.** 5564 DEGs between two TRP-clusters.

**Supplementary Table 4**. 4605 DEGs between tumor and normal tissue.

**Supplementary Table 5**. 1329 genes were associated with OS based on univariate Cox regression analysis.
